# Interdisziplinäre Zentren für Autoimmunerkrankungen in Deutschland

**DOI:** 10.1007/s00393-024-01542-7

**Published:** 2024-07-25

**Authors:** Margitta Worm, Claudia Günther, Martin Claussen, Gernot Keyßer, Ina Kötter, Gabriela Riemekasten, Elise Siegert, Norbert Blank, Cord Sunderkötter, Gabriele Zeidler, Peter Korsten

**Affiliations:** 1 Abteilung für Allergologie und Immunologie, Klinik für Dermatologie, Venerologie und Allergologie, Charité Universitätsmedizin Berlin, Charitéplatz 1, 10117 Berlin, Deutschland; 2 Klinik und Poliklinik für Dermatologie, Universitätsklinikum Carl Gustav Carus, TU Dresden, Dresden, Deutschland; 3 Abteilung Pneumologie, LungenClinic Grosshansdorf, Grosshansdorf, Deutschland; 4 Arbeitsbereich Rheumatologie, Universitätsklinik und Poliklinik für Innere Medizin II (Nephrologie, Rheumatologie, Endokrinologie/Diabetologie), Universitätsklinikum Halle (Saale), Halle, Deutschland; 5 Klinik für Rheumatologie und Immunologie Bad Bramstedt und Sektion Rheumatologie, III. Medizinische Klinik und Poliklinik (Nephrologie/Rheumatologie/Endokrinologie), Zentrum für Innere Medizin, Universitätsklinikum Hamburg-Eppendorf, Hamburg-Eppendorf, Deutschland; 6 Klinik für Rheumatologie und klinische Immunologie, Campus Lübeck, Universitätsklinikum Schleswig-Holstein, Lübeck, Deutschland; 7 Medizinische Klinik mit Schwerpunkt Rheumatologie und Klinische Immunologie, Charité Universitätsmedizin Berlin, Berlin, Deutschland; 8 Medizinische Klinik V, Sektion Rheumatologie, Zentrum für Innere Medizin, Universitätsklinikum Heidelberg, Heidelberg, Deutschland; 9 Universitätsklinik und Poliklinik für Dermatologie und Venerologie, Universitätsklinikum Halle (Saale), Halle, Deutschland; 10 Klinik I – Internistische Rheumatologie, Osteologie und spezielle Schmerztherapie, Johanniter Krankenhaus Treuenbrietzen, Treuenbrietzen, Deutschland; 11 Klinik für Rheumatologie und Klinische Immunologie, St. Josef-Stift Sendenhorst, Sendenhorst, Deutschland

**Keywords:** Therapiestandard, Leitlinien, Spezialärztliche Versorgung, Systemische Sklerose, Standard of care, Guidelines, Medical specialist care, Systemic sclerosis

## Abstract

**Hintergrund:**

Die Betreuung von Patient:innen mit komplexen Autoimmunerkrankungen erfordert eine interdisziplinäre medizinische Versorgung. In Deutschland gibt es zwar eine zunehmende Anzahl von interdisziplinär arbeitenden Zentren für Autoimmunerkrankungen, jedoch sind sie noch nicht flächendeckend vorhanden und ihre Schwerpunkte und interdisziplinären Strukturen häufig nicht nach einem allgemein konsentierten Standard organisiert. Ferner sind sie bislang nicht regelhaft in der allgemeinen Versorgungsstruktur abgebildet.

**Ziel der Arbeit:**

Die Versorgungsstruktur für Autoimmunpatient:innen am Beispiel eines etablierten universitären Zentrums und eines klinischen Falls wird analysiert.

**Material und Methoden:**

Um exemplarisch eine Standortbestimmung interdisziplinär arbeitender Autoimmunzentren in Deutschland durchzuführen, wurden ein Universitätsklinikum für die Strukturanalyse sowie eine Fallvorstellung zur Betrachtung der klinischen Betreuung ausgewählt.

**Ergebnisse:**

In dem ausgewählten Universitätsklinikum werden Patient:innen mit Autoimmunerkrankungen durch Expert*innen verschiedener Fachdisziplinen interdisziplinär betreut. Die Strukturen sind in einem Organigramm verankert. Mithilfe standardisierter Diagnostik- und Therapiepfade („standard operating procedures“ [SOP]) werden Maßnahmen, die für eine jeweils umfassende Diagnostik und Therapie bestimmter Autoimmunerkrankungen fachübergreifend nötig sind, festgelegt. Der von uns vorgestellte Fall stellt anhand einer Patientin mit systemischer Sklerose und Lungenbeteiligung dar, wie ein standardisierter diagnostischer und therapeutischer Pfad in der Praxis umgesetzt werden kann.

**Diskussion:**

Wir diskutieren, welche Maßnahmen fachübergreifend für eine umfassende Diagnostik und Therapie bestimmter Autoimmunerkrankungen notwendig sind, welche Herausforderungen sich bei der Umsetzung ergeben und welche Vorteile sich gegenüber Leitlinien ergeben können – unter anderem weil sie sofort an neue Erkenntnisse angepasst werden können. Die Etablierung eines nationalen Konsenses für den Aufbau, die erforderlichen Strukturen und die Umsetzung in der Patient:innenversorgung innerhalb interdisziplinär arbeitender Zentren für Autoimmunerkrankungen in Deutschland ist wünschenswert.

## Abstract (en)

**Background:**

Interdisciplinary medical treatment is required to care for patients with complex autoimmune diseases. Although there are an increasing number of interdisciplinary centers for autoimmune diseases in Germany, they are not yet available throughout the country and the focuses and interdisciplinary structures are not organized according to a generally agreed standard. Furthermore, they are not regularly reflected in the general care structure.

**The aim of the work:**

To analyze the care structure using as an example an established center and a clinical case to demonstrate the usefulness of in-house standardized procedures.

**Material and methods:**

In order to determine the status quo regarding interdisciplinary centers for autoimmune diseases in Germany, a university hospital is exemplarily presented for a structural analysis and a case presentation from another center to demonstrate the importance of an interdisciplinary patient care.

**Results:**

At the selected center for autoimmune diseases of the university hospital, patients with autoimmune diseases receive interdisciplinary care from experts from various disciplines. The structures are anchored in an organizational chart. The case report demonstrates a standardized diagnostic and therapeutic pathway (standardized operating procedures, SOP) in a patient with systemic sclerosis and lung involvement.

**Discussion:**

The article discusses which measures are necessary across disciplines for comprehensive diagnostics and treatment of certain autoimmune diseases, which challenges arise during implementation and which advantages can arise compared to guidelines because, among other things, they can be immediately adapted. The establishment of a national consensus for the structure, necessary settings and implementation into patient care within an interdisciplinary center for autoimmune diseases is desirable.

Die Betreuung von Patient:innen mit komplexen Autoimmunerkrankungen mit verschiedenen Organbeteiligungen erfordert eine interdisziplinäre medizinische Versorgung. Das betrifft in der Rheumatologie beispielsweise die Kollagenosen wie den systemischen Lupus erythematodes, die Dermatomyositis und die systemische Sklerose (SSc). Diese Erkrankungen gehören mit weniger als 5 Betroffenen auf 10.000 Einwohner zu den seltenen Erkrankungen und benötigen bei der Betreuung eine spezielle fachliche Expertise und Organisationsstruktur.

Die SSc ist eine Autoimmunerkrankung letztlich ungeklärter Ätiologie, die durch eine Haut- und Organfibrose sowie eine Vaskulopathie gekennzeichnet ist [1]. Klinisch stellen sich verschiedene Formen (limitiert, diffus, sine Scleroderma, Overlap-Formen) dar, die sich in Form und Ausmaß der Organbeteiligungen unterscheiden [1, 7]. Neben der Untersuchung der Haut nach Skleroseausprägung, Ausbreitung, Verteilungstyp und Teleangiektasien muss auch nach möglichen Komplikationen wie den digitalen Ulzerationen (DU) oder einer Calcinosis cutis gesucht werden. Darüber hinaus sind regelmäßige Untersuchungen innerer Organe z. B. hinsichtlich der inzwischen mortalitätsbestimmenden Lungenbeteiligung (interstitielle Lungenerkrankung, „interstitial lung disease“ [ILD]) sowie der pulmonalarteriellen Hypertonie (PAH) erforderlich.

In Deutschland erfasst das Deutsche Netzwerk Systemische Sklerodermie e. V. (DNSS) systematisch betroffene Patient:innen mit einer systemischen Sklerose in einer prospektiven Registerstudie [13]. Auf europäischer Ebene trägt die *European Scleroderma Trials & Research Group* (EUSTAR) ebenfalls mittels eines Registers und verschiedener Projekte dazu bei, die Versorgung von SSc-Patient:innen zu verbessern [3, 9].

Beide Register sind aus einer interdisziplinären Zusammenarbeit hervorgegangen. In Deutschland gibt es nur wenige ausdrücklich als Autoimmunzentren (AIZ) deklarierte Kliniken, die ihre interdisziplinäre Zusammenarbeit in Form von Verfahrensanweisungen und Konferenzen standardisiert haben. Solche AIZ findet man beispielsweise in Kiel, Bonn, Frankfurt, Mainz, Mannheim, Heidelberg, Freiburg, Berlin (Charité), Lübeck, Dresden, Erlangen oder Tübingen. Sie sind bisher primär an Universitätskliniken angesiedelt. Die klinischen Schwerpunkte und interdisziplinären Strukturen sind zumeist individuell organisiert und bislang in der allgemeinen Versorgungsstruktur komplex oder unzureichend aufgestellt.

Interdisziplinär arbeitende AIZ sind zum Teil mit Entzündungszentren [15] und mit Zentren für seltene Erkrankungen (ZSE) vernetzt, die seit 2009 in Deutschland etabliert wurden. Interdisziplinäre AIZ können Teil der als sog. B‑Zentren in ZSE assoziiert sein.

Im Folgenden möchten wir exemplarisch das Dresdener Autoimmunzentrum darstellen und beispielhaft den Nutzen, aber auch die Herausforderungen, die die Arbeit eines solchen Zentrums mit sich bringen, aufzeigen. Zum anderen wollen wir am Beispiel der SSc und anhand eines standardisierten Diagnostik- und Therapiepfades aufzeigen, welche Bedeutung SOPs (Standardisierte Diagnostik- und Behandlungspfade) und die Durchführung von interdisziplinären Fallkonferenzen im Kontext von Autoimmunzentren für die Betreuung von Patient:innen mit seltenen Erkrankungen haben.

## Methoden

Die Autorengruppe setzt sich interdisziplinär zusammen (Dermatologie, Rheumatologie, Pneumologie), umfasst einzelne Zentren des DNSS aus der Region Nord-Ost (Lübeck, Hamburg, Göttingen, Berlin, Halle, Dresden und Treuenbrietzen in Deutschland und Herrn Prof. Blank als Vorsitzenden des DNSS e. V.) und hat sich wiederholt ausgetauscht, um die Thematik universitärer AIZ zu diskutieren. Ziel dieser Diskussion war es, den Status quo exemplarisch zu analysieren, darauf aufbauend Barrieren für die Umsetzung zu identifizieren und Empfehlungen für eine erfolgreiche Etablierung von AIZ zu erarbeiten (Tab. 1).

**Tab. 1:** Aufgaben, Anforderungen und Vorteile von interdisziplinär arbeitenden Autoimmunzentren mit dem Ziel der Messbarmachung von Versorgungsstrukturen

Aufgaben	Anforderungen	Vorteile
– Verbesserte Patient:innenversorgung – Verkürzte Wartezeiten – Verkürzte Zeit bis zur Diagnosestellung – Frühere Therapieeinleitung – Definition von Kriterien für interdisziplinäre Konferenzen	– Infrastrukturelle Ressourcen (Personal und Räume) – Interdisziplinäre Präsenz – Abbildung der Vergütung ambulanter Leistungen – Antragstellung bei „off-label“-Therapie – Erstellung von Diagnostik- und Behandlungspfaden	– Datenbanken für klinische Studien – Aufbau klinisch gut definierter Kohorten mit Biobanking – Etablierung von Forschungsverbünden – Fachübergreifende medizinische Patient:innenbetreuung

## Ergebnisse

### Das interdisziplinäre Autoimmunzentrum am Universitätsklinikum Dresden

**Abb. 1:**
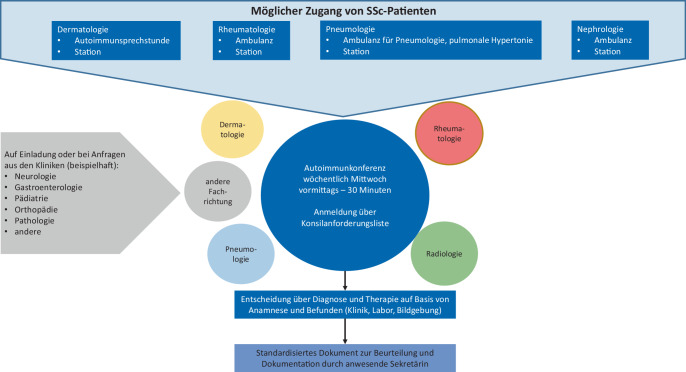
Übersicht zum Konzept eines Zentrums zur Betreuung von Patient:innen mit systemischer Sklerose (SSc)

Im Jahr 2016 wurde das UniversitätsCentrum für Autoimmun- und Rheumatische Erkrankungen am Universitätsklinikum Dresden gegründet. Es dient der fachlichen Vernetzung und Verbesserung der Zusammenarbeit der unterschiedlichen klinischen Fachbereiche, die primär Patienten mit Autoimmun- und verwandten Erkrankungen behandeln. Dazu zählen die Fachbereiche Rheumatologie, Dermatologie, Nephrologie, Pneumologie, Gastroenterologie, Neurologie, Ophthalmologie und pädiatrische Rheumatologie (Abb. 1).

Fallkonferenzen wurden eingerichtet, die speziell für Patient:innen mit seltenen schwerwiegenden Autoimmunerkrankungen, organbedrohenden Komplikationen oder komplexen Therapieverfahren vorgesehen sind. Im Unterschied zum regulären Konsiliarsystem erfolgt eine gemeinsame Besprechung eines Falles durch Ärzt:innen der verschiedenen Disziplinen mit dem Ziel, weitere Diagnostik und Therapieverfahren festzulegen. Das Ergebnis der Beratung wird als Empfehlung dokumentiert, die für alle Mitglieder des UniversitätsCentrum für Autoimmun- und Rheumatische Erkrankungen einsehbar ist. Neben dem Bericht sind für alle beteiligten Ärzt:innen auch die Laborwerte und Befunde der Patient:innen aus anderen Fachbereichen einsehbar, die sonst aus datenschutzrechtlichen Gründen gesperrt wären.

Je nach Formenkreis der Erkrankung bzw. führender Organsymptomatik erfolgen Fallkonferenzen z. B. Kollagenose- und Vaskulitis-Konferenz mit obligater Beteiligung der Rheumatologie – Dermatologie – Nephrologie – Pneumologie und den übrigen Disziplinen nach Bedarf auf Einladung optional, der neuroradiologisch-neuroimmunologischen Konferenz (Neuroradiologie – multiple Sklerose – Zentrum) und der radiologisch-rheumatologischen Konferenz (Radiologie – Rheumatologie). Die Fallkonferenzen finden je nach Patient:innenaufkommen wöchentlich bis monatlich statt. Während der Konferenzen werden Patient:innen von den betreuenden Ärzt:innen anhand der elektronischen Krankenakte vorgestellt. Das weitere diagnostische Vorgehen und die möglichen Therapieoptionen werden interdisziplinär diskutiert. Voraussetzung für die Vorstellung von Patient:innen in der Fallkonferenz ist, dass sie in einer der Spezialambulanz des Klinikums betreut oder aktuell stationär behandelt werden.

Weitere monatliche Zusammenkünfte mit optionaler gemeinsamer Patient:innenvorstellung bieten fachübergreifende Strukturen wie die ophthalmologisch-immunologische Konferenz (Ophthalmologie – Rheumatologie – Kinderrheumatologie – Neurologie), die Psoriasisarthritis-Sprechstunde (Dermatologie – Rheumatologie), die rheumaorthopädische Sprechstunde (Orthopädie – Rheumatologie) und die Transitionssprechstunden chronisch entzündliche Darmerkrankungen (CED, [Kindergastroenterologie-CED-Ambulanz]) und Rheuma (Kinderrheumatologie-Rheumatologie).

Neben den interdisziplinären Fallkonferenzen ist ein weiteres Ziel des Zentrums die Standardisierung fachübergreifender Behandlungsverfahren, wie z. B. Rituximab- oder Cyclophosphamid-Infusionen. Dafür werden Therapiepläne („standard operating procedures“ [SOPs]) erstellt und zwischen den Fachabteilungen abgestimmt, die in einem zentralen Benutzerhandbuch für alle teilnehmenden Abteilungen einsehbar sind (Gültigkeit in der Regel 2 Jahre).

Das Zentrum steht für Anfragen niedergelassener Fachärzt:innen und anderer Krankenhäuser zur Verfügung. Als Plattform dient hierfür ein Gesamtboardmeeting, das alle 2 Monate stattfindet. Externe Kolleg:innen können selbst Fälle präsentieren oder um die Diskussion ihres Falles bitten. Das AIZ ist dem ZSE angegliedert, sodass Anfragen von Fachärzt:innen oder Patient:innen an das ZSE zu Autoimmun- und Rheumaerkrankungen an die Ärzte des AIZ weitergeleitet werden.

Von dieser Struktur profitieren Patient:innen mit Erkrankungen, die regelhaft mehrere Organsysteme betreffen, beispielsweise solche mit systemischer Sklerose, für die die Betreuung im Folgenden exemplarisch dargestellt wird:

Je nach vorherrschender Symptomatik (Haut, Lunge, Niere) werden die Patient:innen mit Verdacht oder bereits diagnostizierter systemischer Sklerose meist von niedergelassenen Fachärzt:innen an die Spezialambulanzen des Universitätsklinikums überwiesen (Abb. 1). Dort wird dann die Organdiagnostik koordiniert. Praktisch erfolgt dies durch Terminvergabe und Vorstellung in den anderen Fachbereichen. Kommt ein:e Patient:in z. B. primär in die Dermatologie, werden eine Lungenfunktionsuntersuchung und eine Echokardiographie veranlasst. Sobald alle Befunde vorliegen, werden sie von Ärzt:innen der primär behandelnden Spezialambulanz in der wöchentlich stattfindenden Kollagenose- und Vaskulitis-Konferenz zusammengefasst und vorgestellt. Gemeinsam wird z. B. das Vorgehen bei Fortschreiten der Erkrankung z. B. der interstitiellen Lungenerkrankung festgelegt, schriftlich fixiert und den niedergelassenen Kolleg:innen übermittelt (Abb. 1). In der Besprechung kann auch zusätzlich eine begleitende Mitbetreuung anderer Spezialambulanzen vereinbart werden. So können beispielsweise primär in der Dermatologie vorgestellte Patient:innen Zugang zu einer neurologischen oder rheumatologischen Mitbetreuung am Universitätsklinikum erhalten, wenn diese nicht durch niedergelassene Kolleg:innen gewährleistet oder aufgrund des erhöhten Versorgungsbedarfes an der Universitätsklinik sinnvoll ist. Dieses Konzept der interdisziplinären Versorgung verbessert die Behandlung der Patient:innen und erleichtert auch deren Zugang zu innovativen Therapien im Rahmen klinischer Studien, die in den verschiedenen Spezialambulanzen angeboten werden. Der Zugang zu Befunden aller Spezialambulanzen erleichtert zudem die Dokumentation in Registerstudien wie dem DNSS (Deutsches Netzwerk für Systemische Sklerodermie) oder EUSTAR (European Scleroderma Trials and Research Group) und verbessert dadurch langfristig die Betreuung und Versorgung der Patient:innen.

Neben der oben exemplarisch dargestellten strukturellen Sicherstellung einer interdisziplinären Versorgung ist die Umsetzung standardisierter Diagnostik- und Therapiepfade für ein modernes Patient:innenmanagement essenziell. Obgleich die Implementierung von Leitlinien hier eine zentrale Bedeutung hat, ist festzustellen, dass diese nicht immer für seltene Erkrankungen existieren bzw. sich deren Überarbeitung mit aktuellen medizinischen Entwicklungen (neue Therapien) nicht selten verzögert. Dieser Situation wird in universitären Zentren, wie oben beschrieben, mit einer klinik- und oder zentrumsweiten Implementierung von SOPs begegnet, die wir im folgenden Fallbericht darstellen und diskutieren möchten.

## Notwendigkeit der Entwicklung von SOPs – Beispiel SSc-ILD

### Fallbeispiel

Eine 42-jährige Patientin wurde dem Zentrum zur Organdiagnostik und Therapieeinleitung bei Erstdiagnose einer SSc-ILD zugewiesen. Die Diagnose basierte auf den klinischen Befunden einer klinisch diffusen Sklerodermie (modifizierter Rodnan Skin Score 26 von maximal 51 Punkten) und eines sekundären Raynaud-Syndroms seit 3,5 Jahren, einer Sklerodaktylie, Teleangiektasien, einer gastroösophagealen Refluxerkrankung und den serologischen Befunden hochpositiver antinukleärer Antikörper sowie Anti-Topoisomerase I (Scl-70)-Antikörper. Weiterhin zeigte sich in der Kapillarmikroskopie ein „active pattern“ nach Cutolo mit Megakapillaren und Hämorrhagien.

Es stellen sich folgende Fragen:

1. Welche weitere Diagnostik ist im vorliegenden Fall notwendig?
2. Welche Therapie(n) sollte(n) eingeleitet werden?

Bei der SSc liegen die Hauptursachen für eine im Vergleich zur Allgemeinbevölkerung erhöhte Mortalität in der möglichen Entwicklung einer interstitiellen Lungenerkrankung (ILD) oder einer pulmonalarteriellen Hypertonie (PAH) mit konsekutiver Rechtsherzinsuffizienz [1]. Daher ist ein standardisiertes Vorgehen der Diagnostik und Therapie bei einer SSc erforderlich. Hierbei spielen Leitlinien eine zentrale Rolle, und so sind z. B. die Empfehlungen der europäischen rheumatologischen Fachgesellschaft [12] sowie ein europäisches Konsensuspapier für die Diagnostik und Therapie der SSc-ILD verfügbar [10]. Da Leitlinien sich aufgrund der Vorgaben für die Qualität und Bewertung der Datenlage immer streng am Evidenzniveau orientieren müssen und neuen Entwicklungen durch Überarbeitung nicht immer zeitnah Rechnung tragen können, bieten sich (zentrumsspezifische) standardisierte Vorgehensweisen (SOPs), basierend auf aktuell publizierten Daten und Empfehlungen, an. Diese SOPs haben den Vorteil, dass neuere Studiendaten, noch vor Eingang in Leitlinien, schneller in die klinische Praxis umgesetzt werden können. Es muss jedoch berücksichtigt werden, dass diese zentrumsspezifischen SOPs keine Rechtssicherheit hinsichtlich möglicher Überprüfungen durch Kostenträger bieten. Dennoch kann eine standardisierte Vorgehensweise, gerade bei Dokumentation derselben in interdisziplinären Fallkonferenzen, dabei helfen, gewisse Therapieentscheidungen plausibel nachvollziehbar zu machen.

### Diagnostisches Vorgehen

**Abb. 2:**
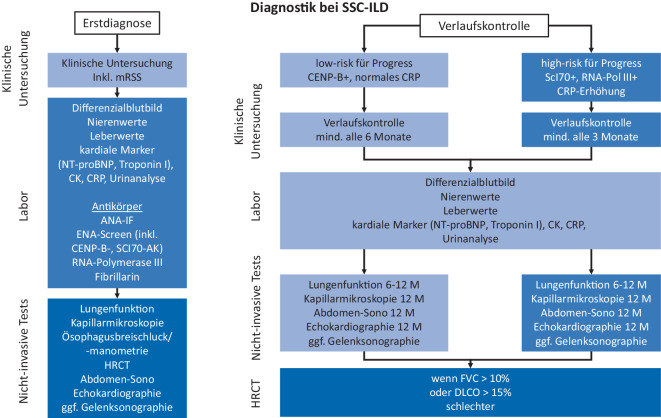
Beispiel einer standardisierten Handlungsanweisung zur Diagnostik bei systemischer Sklerose-assoziierter interstitieller Lungenerkrankung

Neben der vollständigen Anamnese und klinischen Untersuchung einschließlich des modifizierten Rodnan Skin Scores sind laborchemische Analysen und nichtinvasive Untersuchungen erforderlich. Die Abb. 2 zeigt exemplarisch das diagnostische Vorgehen bei Verdacht auf SSc-ILD der Universitätsmedizin Göttingen.

Es muss zwischen der Erstvorstellung und dem Vorgehen bei Verlaufskontrollen unterschieden werden: Bei Erstdiagnose erfolgen neben der Lungenfunktionsprüfung (Spirometrie, Bodyplethysmographie und Bestimmung der Diffusionskapazität) immer auch eine Kapillarmikroskopie der Nagelfalzkapillaren, eine Echokardiographie, eine Abdomensonographie sowie eine hochauflösende Computertomographie (HRCT) der Lunge. Letztere halten wir bei Erstdiagnose in allen Fällen für erforderlich, um die prognosebestimmende SSc-ILD frühzeitig zu entdecken und ggf. auch behandeln zu können.

Während der Verlaufskontrollen sind routinemäßige Untersuchungen mittels HRCT gemäß der Literatur umstritten [11]. Bei Patient:innen mit einem hohen Risiko für eine progrediente Lungenfibrose (z. B. diffus-kutane [dc] SSc, Scl-70-AK, CRP-Erhöhung sowie Fibrose des Lungenparenchyms >20 % bei Baseline) sind kurzfristigere Kontrollen ca. alle 3 Monate mittels klinischer Untersuchung und Lungenfunktion aus Sicht des AIZ Göttingen sinnvoll. Bei weniger hohem Risiko (limitiert-kutane [lc] SSc, CENP-B-AK, keine CRP-Erhöhung) sollten klinische Untersuchungen mindestens alle 6 Monate erfolgen, Lungenfunktionsprüfungen alle 6 bis 12 Monate. Bei vielen Patient:innen kann eine transthorakale Echokardiographie 1‑mal pro Jahr sinnvoll sein, um eine eventuell zusätzlich vorhandene PAH zu entdecken, da es auch hier zugelassene Therapiemöglichkeiten (Phosphodiesterase-5-Hemmer, Stimulatoren der löslichen Guanylatzyklase, Endothelin-Rezeptor-Antagonisten, Prostacyclin-Analoga und Prostacyclin-Rezeptor-Agonisten) und Empfehlungen gibt [8].

Diese Empfehlung wurde in die vorgestellte SOP aufgenommen, auch wenn in anderen Empfehlungen, wie z. B. dem DETECT-Algorithmus, die Notwendigkeit wiederholter Echokardiographien differenzierter gestellt wird. Sofern dies in Zentren nicht möglich ist, kann die Notwendigkeit einer Echokardiographie auch mit dem DETECT-Algorithmus bestimmt werden, um die Vortestwahrscheinlichkeit für eine PAH zu erhöhen [5]. Wiederholte HRCT-Untersuchungen sollten bei einem Abfall der forcierten Vitalkapazität (FVC) von mehr als 10 % oder der Diffusionskapazität für Kohlenmonoxid (DLCO) von mehr als 15 % erfolgen, wobei zunächst auch eine Kontrolle der Lungenfunktionsmessungen z. B. nach 1 bis 3 Monaten sinnvoll sein kann, wenn überlagernde Gründe für die Verschlechterung der Messwerte offensichtlich sind (z. B. schmerzbedingte Einschränkung der Durchführung, kurz zurückliegende Atemwegsinfekte etc.). Bei hohem Risiko für Progress wird von einigen Experten ein Monitoring mittels Lungen-CT empfohlen [11]. Für letzteres Vorgehen gibt es jedoch relativ wenig Evidenz.

### Therapeutisches Vorgehen

Die Therapie der SSc-ILD richtet sich nach dem Ausmaß der Lungenbeteiligung in der Bildgebung der fassbaren Störung der Lungenfunktion, der klinischen Beeinträchtigung, den weiteren Krankheitsmanifestationen sowie dem individuellen Risiko für einen weiteren Progress der Erkrankung.

Eine klinisch praktikable Einteilung in eine subklinische und eine klinisch manifeste ILD wurde von Roofeh et al. vorgeschlagen [14]. Hierbei wurde die subklinische ILD definiert als (1) minimale oder milde Ausdehnung der ILD in der HRCT, (2) normale Lungenfunktion und (3) kein relevanter Abfall der FVC oder DLCO bei wiederholter Lungenfunktionsprüfung.

Die klinisch manifeste ILD hingegen zeigt (1) milde bis schwere Ausprägung im HRCT (> 20 % des Lungenparenchyms), (2) FVC oder DLCO außerhalb des Normbereiches oder (3) signifikanter Abfall der FVC (> 10 %) oder DLCO (> 15 %) im Verlauf.

Therapeutisch kommen im Wesentlichen immunsuppressive Therapien, wie z. B. Mycophenolat-Mofetil (MMF), Ciclosporin A (CsA), Cyclophosphamid (CYC), Tocilizumab (TCZ) oder Rituximab (RTX) sowie antifibrotische Therapieansätze (mit dem für die SSc-ILD zugelassenen Medikament Nintedanib) infrage. Bei progredienten Befunden werden diese Therapien an einzelnen Zentren auch als Kombinationstherapien (z. B. MMF + RTX, MMF + TCZ etc.) eingesetzt, wobei für Letztere keine wissenschaftliche Evidenz oder konkrete Leitlinienempfehlungen existieren. In der nächsten Eskalationsstufe sollte auch eine autologe Stammzelltransplantation in Betracht gezogen werden, für die es mittlerweile positive Daten aus 3 randomisierten kontrollierten Phase-II/III-Studien gibt [4, 17, 18], aber nach aktuellen Metaanalysen noch nicht genügend Daten, um sie allgemein zu empfehlen [16].

**Abb. 3:**
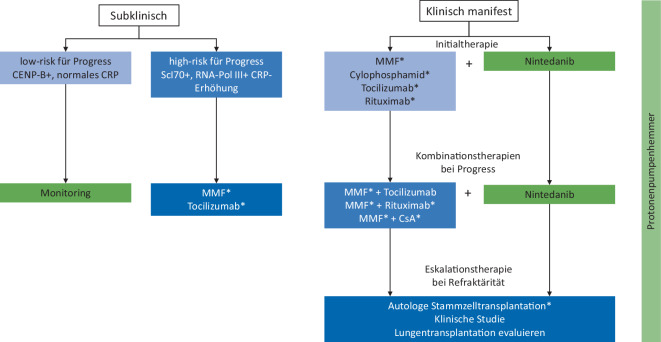
Beispiel einer standardisierten Handlungsanweisung zur Therapie bei systemischer Sklerose-assoziierter interstitieller Lungenerkrankung

Kürzlich konnte eine Publikation aus DNSS-Registerdaten eindrücklich zeigen, dass der Einsatz von Protonenpumpenhemmern bei SSc-ILD vorteilhaft hinsichtlich Mortalität ist [13]. Das therapeutische Vorgehen bei SSc-ILD an der Universitätsmedizin Göttingen ist zusammenfassend exemplarisch in Abb. 3 dargestellt.

Die im Fallbeispiel vorgestellte Patientin wurde entsprechend dem in der SOP festgelegten Vorgehen kombiniert mit Nintedanib sowie Mycophenolat-Mofetil behandelt. Verlaufskontrollen erfolgen regelmäßig ambulant.

## Diskussion

Zahlreiche Autoimmunerkrankungen gehören zu den seltenen Erkrankungen und erfordern aufgrund ihrer Systempathologie mit Beteiligung mehrerer Organsysteme eine interdisziplinäre medizinische Versorgung. Die Versorgungsstrukturen für Patient:innen mit Autoimmunerkrankungen in Deutschland sind bis heute überwiegend fachspezifisch strukturiert. Somit wird eine interdisziplinäre, aber auch altersübergreifende Versorgung betroffener Patient:innen selbst an Universitätskliniken in Deutschland noch nicht in allen Bereichen realisiert.

In der vorgelegten Arbeit stellen wir beispielhaft zum einen ein 2016 gegründetes interdisziplinär arbeitendes universitäres Autoimmunzentrum vor. Im ersten Schritt haben sich die verschiedenen organspezifischen Disziplinen auf eine Zusammenarbeit festgelegt und einen juristischen organisatorischen Rahmen definiert (Zentrumsatzung mit Rechten und Pflichten der teilnehmenden Einrichtungen), der von der Fakultät bzw. der Krankenhausleitung bewilligt wurde. In der Praxis zeigt sich, dass zusätzlich zu den klinischen Spezialisten die Teilnahme diagnostisch spezialisierter Ärzte, insbesondere der Fachrichtung Radiologie und Pathologie, für die gemeinsame therapeutische Planung in einem AIZ notwendig ist. Neben der Auswahl der beteiligten Disziplinen ist die Bereitstellung von räumlichen und personellen Ressourcen erforderlich. Ressourcen zu identifizieren ist für den Träger eines Autoimmunzentrums eine Herausforderung, insbesondere durch spezifische Aspekte der Patient:innenversorgung im Kontext der Diagnostik und Therapie. Gerade bei Autoimmunpatient:innen sind in wiederkehrenden Abständen aufwendige organspezifische Untersuchungen (z. B. CT) erforderlich, die im Rahmen der Hochschulambulanz oder anderer fachspezifischer Ermächtigungen meist nicht hinreichend wirtschaftlich abgebildet werden und deren stationäre Erbringung nicht wirtschaftlich ist. Strukturen eines ambulanten Versorgungszentrums im Sinne einer ambulanten spezialfachärztlichen Versorgung (ASV) sind hilfreich, um diese Versorgungslücken zu schließen. Etablierung ist jedoch von spezifischen medizinischen Voraussetzungen abhängig und bürokratisch aufwendig. Es wird künftig zu prüfen sein, ob das Instrument der ASV für das hier besprochene Konstrukt anwendbar sein könnte oder ob eigene, stärker auf die Belange der Universitätsmedizin zugeschnittene Abrechnungsstrukturen vorteilhafter sind. Da die ambulanten Versorgungszentren für einzelne Krankheiten oder Fachbereiche entwickelt wurden, bilden sie nicht alle an der Behandlung von Autoimmunerkrankungen beteiligten Fachbereiche gleichberechtigt ab bzw. ermöglichen nicht einen dem variablen Krankheitsspektrum angepassten flexiblen Zugangsweg zur Diagnostik. So ist beispielsweise für einen im ASV assoziierten Pneumologen oder Dermatologen keine direkte Abrechnung eines Lungen-CT bei einem Patienten mit systemischer Sklerose möglich. Eine flexible Weiterentwicklung des Systems für universitäre AIZ wäre wünschenswert.

Autoimmunerkrankungen gehören zu den seltenen Erkrankungen. Das Therapiespektrum ist zumeist begrenzt, und nicht selten müssen neue kostenintensive Therapien zum Einsatz kommen, die jedoch im Off-label-Bereich liegen und somit wirtschaftliche Risiken für die verordnenden Ärzte mit sich bringen. Umfassende diagnostische Untersuchungen sind im Verlauf der Betreuung erforderlich, können jedoch kostenintensiv sein. Zudem können zusätzlich auftretende Erkrankungen die Betreuung erschweren (z. B. Hüftoperation bei einem:r Patient:in mit Lungenerkrankung bei SSc). Diese Herausforderungen sollten durch eine direkte Vergütung von Patienten mit seltenen Erkrankungen abgebildet werden. Grundlage für eine entsprechende Erfassung der Fälle ist die Kodierung von Alpha-ID und Orpha-Code, und dies sollte dementsprechend strukturell im Dokumentationssystem der Kliniken ermöglicht werden.

Andererseits bieten interdisziplinär arbeitende medizinische Zentren zahlreiche Vorteile für die „Mutter“-Struktur. Hierzu gehören der Aufbau von Datenbanken für klinische Studien, der Aufbau von Biobanken und die direkte Vernetzung mit der Forschung (Grundlagen und translational). Auch für Patient:innen ergeben sich für die medizinische Versorgung zahlreiche positive Aspekte wie verkürzte Wartezeiten, eine frühzeitige Therapieeinleitung und auch der Einsatz aktueller Diagnostik- und Therapiestandards trotz fehlender oder verzögert verfügbarer Leitlinien. Die standardisierte Erhebung der Diagnostik und Therapie ermöglicht darüber hinaus, eine verbesserte Versorgungsstruktur messbar zu machen.

Das Fallbeispiel aus Göttingen zeigt die Bedeutung einer hinreichenden und v. a. frühzeitigen bildgebenden Diagnostik zur Erkennung einer interstitiellen Lungenerkrankung. Die Mortalität der systemischen Sklerose wird maßgeblich vom Schweregrad und Verlauf der ILD beeinflusst [2]. Speziell zentrumsspezifische Behandlungspfade bzw. SOPs, die aktuelle Entwicklungen des medizinischen Fortschritts berücksichtigen, kamen hier zur Anwendung.

MMF, das trotz guter Evidenz gemäß publizierter Studien [14] nach wie vor nur im Off-label-Bereich zur Behandlung einer systemischen Sklerose eingesetzt werden kann, wurde bei dieser Patientin mit dem erst kürzlich zugelassenen Medikament Nintedanib kombiniert [6].

Andere Therapieoptionen wären eine kombinierte Behandlung mit anderen Immunsuppressiva wie CsA oder Cyclophosphamid bzw. Biologika wie Tocilizumab oder Rituximab [14].

Ob oder welche Medikamentenkombination überlegen sein könnte, ist bislang nicht bekannt. Genau eine derartige Fragestellung wäre höchstwahrscheinlich zügiger zu beantworten, wenn Patienten primär ausschließlich in Autoimmunzentren betreut würden und diese darüber hinaus vernetzt zusammenarbeiten würden. Ein erster Ansatz wurde mit dem deutschen Sklerodermie-Netzwerk (DNSS) realisiert, wo Patienten mit systemischer Sklerose standardisiert erfasst und zum Teil prospektiv beobachtet werden. Hier wurden erstmals Daten von Patienten mit systemischer Sklerose in Deutschland erfasst, die zu einem besseren Verständnis der Erkrankung beigetragen haben.

Das DNSS könnte zukünftig eine wichtige Rolle für die Weiterentwicklung und Aufstellung von Autoimmunzentren spielen, beispielsweise durch die Festlegung von Qualitätsstandards und Zertifizierungen.

Zusammenfassend möchten wir mit unserer Arbeit darauf hinweisen, dass es in Deutschland zwar einige interdisziplinär arbeitende Autoimmunzentren an deutschen Universitätskliniken gibt, jedoch in zahlreichen Bundesländern noch Entwicklungspotenzial besteht, um die Patient:innen auf höchstem Niveau zu versorgen. Neben strukturellen und wirtschaftlichen Barrieren, die überwunden werden müssen, bestehen hohe Potenziale für die Versorgung von Patient:innen, aber auch die translationale Forschung.
